# In Search of the Most Relevant Parameter for Quantifying Lung Inflammatory Response to Nanoparticle Exposure: Particle Number, Surface Area, or What?

**DOI:** 10.1289/ehp.9254

**Published:** 2006-10-03

**Authors:** Klaus Wittmaack

**Affiliations:** GSF – National Research Center for Environment and Health, Institute of Radiation Protection, Neuherberg, Germany

**Keywords:** joint length, lung inflammation, particle mass, particle number, saturation effects, specific surface area, ultrafine carbon particles

## Abstract

**Background:**

Little is known about the mechanisms involved in lung inflammation caused by the inhalation or instillation of nanoparticles. Current research focuses on identifying the particle parameter that can serve as a proper dose metric.

**Objectives:**

The purpose of this study was to review published dose–response data on acute lung inflammation in rats and mice after instillation of titanium dioxide particles or six types of carbon nanoparticles. I explored four types of dose metrics: the number of particles, the joint length—that is, the product of particle number and mean size—and the surface area defined in two different ways.

**Findings:**

With the exception of the particle size–based surface area, all other parameters worked quite well as dose metrics, with the particle number tending to work best. The apparent mystery of three equally useful dose metrics could be explained. Linear dose–response relationships were identified at sufficiently low doses, with no evidence of a dose threshold below which nanoparticle instillation ceased to cause inflammation. In appropriately reduced form, the results for three different sets of response parameters agreed quite well, indicating internal consistency of the data. The reduced data revealed particle-specific differences in surface toxicity of the carbon nanoparticles, by up to a factor of four, with diesel soot being at the low end.

**Conclusions:**

The analysis suggests that the physical characterization of nanoparticles and the methods to determine surface toxicity have to be improved significantly before the appropriate dose metric for lung inflammation can be identified safely. There is also a need for refinements in quantifying response to exposure.

Possible adverse health effects due to the inhalation of airborne particulate matter (PM) are a topic of ongoing scientific and public concern (Lippmann et al. 2003). Recently, attention has focused on the effect of particles with sizes < 100 nm, referred to as ultrafine particles (Brown et al. 2000) or nanoparticles (Nel et al. 2006; Oberdörster et al. 2005). Interest in ultrafine particles is due to the fact that, on the basis of the toxicity of the various chemical compounds contained in ambient PM, the epidemiologically identified associations between adverse health effects and PM are not plausible (Green et al. 2002; Valberg 2004). Hence, one needs to explore the idea that the mere physical presence of insoluble nanoparticles deposited deep in the lung may cause adverse effects, including the possibility that these small particles may enter into the blood circulation and are subsequently translocated to sensitive body organs such as the liver, the heart, or even the brain (Oberdörster et al. 2005).

The toxic potential of insoluble nanoparticles is commonly explored in laboratory studies involving rats or mice. A frequently studied response parameter is lung inflammation. *In vivo* studies have aimed at determining the (physical) parameter that can serve as a relevant measure of the applied dose. The knowledge derived from such studies may pave the way to identifying mechanistic pathways of lung inflammation. Previous work has shown that, for the same dose in terms of mass, response increases with decreasing particle size. To explain these observations, investigators have considered the surface area as the proper dose metric (Donaldson et al. 2000; Duffin et al. 2002; Oberdörster 1996, 2000; Oberdörster et al. 2005; Stoeger et al. 2006). One of the problems in such studies is that nanoparticles made from different materials exhibit large differences in inflammogenic potential. The surface toxicity was found to be low for carbon, titanium dioxide, and latex but very high for quartz, cobalt, and nickel (Duffin et al. 2002). The origin of these differences is not known.

Another problem with the previous studies involving particles of low surface toxicity is that high doses were often used in order to observe a significant response. Hence, heavily nonlinear effects were observed quite frequently, without being acknowledged as such (Oberdörster 1996, 2000; Stoeger et al. 2006). Linear dose–response relationships have also been reported, but more rarely (Duffin et al. 2002). The issues of lung overload and maximum tolerated doses have previously been discussed with reference to particle-induced carcinogenesis (Mauderly 1996; McConnell 1996). In the nonlinear dose range the inflammogenic potential of nanoparticles cannot be quantified accurately. Furthermore, the dose–response relationship observed in the nonlinear dose range could depend on parameters other than the proper dose metric so that identification of the latter may become difficult, if not impossible.

Most of the previous work on lung inflammation has focused on exposure involving fine and ultrafine insoluble particles in the size range between 20 and 250 nm. Response to particles with sizes < 20 nm has been explored only recently (Stoeger et al. 2006). All of the studies had in common that conversion of particle masses to surface areas was based on the method developed by Brunauer, Emmett, and Teller (BET; Brunauer et al. 1938). In a BET analysis the specific surface area *S*—the ratio *A*/*M* of the surface area *A* to the mass *M* of particles—is derived from the particle’s gas absorption characteristics. The method has become popular, partly because of the lack of alternative techniques. Until now, basic studies providing a direct justification for the use of the BET surface as a dose metric in lung inflammation have not been presented. The common approach has been to explore whether response data measured with particles of different size exhibit a common trend when plotted as a function of the BET surface area of the instilled particles.

It is important to note that in the size range < 20 nm, proper characterization of particle parameters becomes difficult. In addition to the BET surface area, the particle morphology, the porosity, and the agglomeration state are of key interest. Standard transmission electron microscopy (TEM) or scanning electron microscopy (SEM) may be used to achieve preliminary insight into particle agglomeration and changes due to particle transfer into aqueous solutions (Bérubé et al. 1999), such as those used for instillation. Detailed information on the structure of bonds between agglomerated particles can be obtained only by high-resolution transmission electron microscopy (HRTEM) (Ishiguro et al. 1997), a technique that has not yet been employed to characterize nanoparticles for use in exposure studies.

One should also note that there is no simple strategy for quantifying lung inflammation in a straightforward manner. A common approach to determining inflammatory response is to measure the number of polymorphonuclear leukocytes (PMNs) found in the bronchoalveolar lavage (BAL) at some time after exposure. A problem with this is the identification of the most appropriate time interval between exposure and lavage. Another problem is whether and to what extent the lavageable cells constitute a sufficiently accurate measure of response at all applied doses. Furthermore, the PMNs generated in response to particle exposure are somehow in competition with macrophages and other cells. Hence, it becomes debatable, notably at doses where the PMNs and the other cells are similar in number, whether the response should be quoted in terms of the total number of PMNs, as a ratio or a concentration (fraction). With these inherent complications in mind, my aim in this study was to significantly extend previous attempts at identifying the appropriate dose metric in lung inflammation by fine and ultrafine particles.

## Data and Methods

Two sets of lung inflammation data were considered, both involving exposure by instillation. The first study concerned 20- and 250-nm titanium dioxide (TiO_2_) particles in rats (Oberdörster et al. 2000). The BET-specific surface areas were not quoted explicitly but could be derived from the data in the respective figures (50 and 6.5 m^2^/g, respectively; specifications presumably supplied by the particle manufacturer). The particles appear to have been the same as those used by another group in a study on a similar topic (Donaldson et al. 2000). The PMNs in the bronchoalveolar lavage were measured 24 hr after exposure. Data analysis in terms of the instilled BET surface area suggested virtually identical inflammatory response to the TiO_2_ particles of different size.

In the second study (Stoeger et al. 2006), six different types of ultrafine carbon particles were instilled in mice. The BET-specific surface areas ranged from about 35 to 800 m^2^/g, the mean particle sizes (by TEM) from 10 ± 2 to 45 ± 15 nm, and the organic carbon levels between 1 and 20%. Most of the parameters were determined by the authors or at their request. The response parameters included the number of PMNs and—in the BAL fluid (BALF)—the concentrations of cytokines, notably interleukin 1β (IL-1β), as well as the concentrations of the macrophage inflammatory protein 2 (MIP2). The authors concluded that the levels of inflammatory response to carbon particles were *a*) dependent on particle type and mass, *b*) most strongly related to the BET surface area, and *c*) became evident only if the surface area of the instilled particles exceeded a “threshold” of about 20 cm^2^.

The data discussed below were adapted from the figures in the original papers. Statistical analysis was performed using the graphics package ORIGIN 5.0 (Microcal, Northampton, MA, USA).

## Results and Discussion

**Figure 1:**
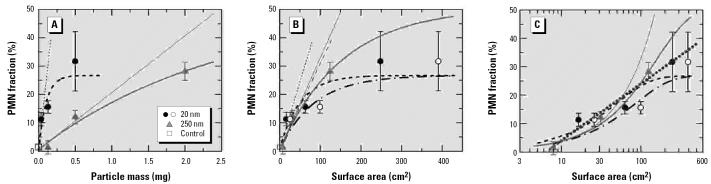
Neutrophil fraction measured in the BAL of rats in response to instillation of titanium dioxide particles of two different sizes versus particle mass (*A*) and the surface area of the particles (*B,C*). Adapted from Oberdörster et al. (2005). The fit functions according to Equations 1 and 2 are denoted by thick and thin lines, respectively. In *B* and *C* the solid circles relate to the BET-based surface area and the open circles to the surface area derived from the particle size. Note the logarithmic *x*-axis in *C*. Data are mean ± SD.

The first example, presented in Figure 1A, shows the PMN fraction (percentage) in BAL of rats due to intratracheal instillation of TiO_2_ particles (Oberdörster 2000; Oberdörster et al. 2005). The results are presented as a function of the applied particle mass. Two observations are noteworthy: *a*) As already stated in the original work, for the same dose in terms of mass *M*, there is a large difference in inflammatory response to particles of different size. *b*) For 250-nm particles, a linear dose response appears to hold for *M* ≤ 0.5 mg. For 20-nm particles, however, linearity in response is less evident and, if present, limited *M* ≤ 50 μg.

The measured data can be described by response functions *R*(*M*) of the type


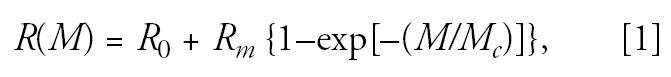


where *R*_0_ is the zero dose response, commonly determined in control experiments, *R_m_* is the assumed maximum exposure–related response and *M_c_* is a characteristic mass. In the limit of small masses *R*(*M*) features a linear dose–response relationship. For *M* << *M_c_*,


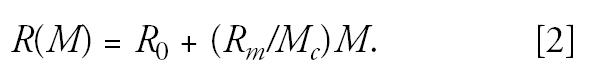


The ratio *R_m_*/*M_c_* may be referred to as a linear response rate. It should be noted that, to be useful, the chosen type of response function must be valid for all particle sizes. Otherwise a search for the proper dose metric would rely on an arbitrary interpretation of experimental data.

Taking into account *R*_0_, the three data points per particle size, and the error bars, the fit parameters *R_m_* and *M_c_* were varied until best agreement was obtained by visual inspection. The derived fit functions are shown in Figure 1A as thick lines, the (extrapolated) linear section as thin lines.

The same data are presented in Figure 1B as a function of the BET surface area *A*, with *A* = *S_BET_M*. The adequacy of the fit functions in the region of small surface areas is evident from Figure 1C. In Figure 1B the presumably linear sections of the dose–response curves for the data—represented by solid symbols—exhibit distinctly different slopes—represented by the dashed and solid straight lines. The differences depend on the BET input data. Hence, one might wonder how the specific surface areas, *S_BET_*, compare with the numbers calculated under the assumption that the particles were spherical. For particles of diameter *D* and mass densityρ, we have the simple relation


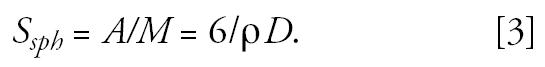


With ρ = 3.9 g/cm^3^ for the anatase modification of TiO_2_, one finds *S_sph_*(20 nm) = 77 and *S_sph_*(250 nm) = 6.2 m^2^/g. The latter result is in good agreement with *S_BET_* = 6.5 m^2^/g. For the 20-nm particles, on the other hand, *S_BET_* = 50 m^2^/g amounts to only 65% of the calculated value. This large difference is hard to understand because any deviation from a smooth spherical form, such as large roughness or high porosity, would make the surface area larger not smaller than the calculated value.

It is worth noting that for a quantitative comparison with calculated specific surface areas *S_cal,_* the assumption of an exact spherical shape of the particles is not required. In fact, the specific surface area of a cube has exactly the same form as Equation 3, provided the parameter *D* is read as the side length of the cube. Hence, a straightforward assessment of the surface area does not necessarily require particles of spherical shape. It suffices to have compact particles with similar size in all three cartesian directions.

Formal agreement between *S_BET_* and the calculated specific surface areas of the 20-nm particles might be achieved by assuming that they have the form of cylindrical rods of diameter *D_c_* and length *L_c_*, in which case


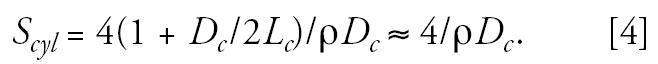


The approximate relation on the right side of Equation 4 holds to within 10% or better for *L_c/_D_c_* ≥ 5. However, it is rather unlikely that TiO_2_ particles in chain agglomerates are so tightly attached to each other that they would appear as (long) rods in the BET analysis (this idea would also be at variance with the trend described below for carbon nanoparticles). Therefore, we must consider the possibility that the BET specification was in error and that the “true” specific surface area of the 20-nm particles was better represented by the calculated value. Results thus obtained are shown in Figure 1B and C as open circles and dash–dotted lines. This alternative mass-to-area conversion causes the initial gradients for the two types of particles to agree quite well, a finding that may be read as saying that the surface area is an appropriate dose metric, provided we use the “best” method for quantifying this parameter. However, a consequence of making recourse to the calculated value of the specific surface area would be that the response observed with particles of distinctly different size tends to diverge in the nonlinear dose range, more so the larger the surface area. Hence, I reach the conclusion, already indicated in the data derived from the BET-based mass-to-surface conversion, that measurements outside the linear dose range are not suited for testing a certain particle parameter with respect to its use as a dose metric.

Data presented in Figure 1C, with the surface area on a logarithmic scale, can serve as a good example for discussing the possible presence of a threshold in a dose–response relationship (Oberdörster et al. 2005). If experimental data are presented in only this way, as in a previous study showing the neutrophil response after exposure of young rats to inhaled endo-toxin (Oberdörster 2000), one might mistakenly conclude that the response is logarithmic, as indicated by the straight dotted line. The line suggests a threshold of about 7 cm^2^, but this interpretation is incorrect because one is not realizing that in a log-linear data presentation, a linear dose response appears initially as a very slowly rising curve. This is exemplified by the thin solid line in Figure 1C, which is the same as the thin straight line in Figure 1B. Clearly, data evaluation on linear–linear scales is indispensable when one tries to rationalize measured dose–response data. Only with this method can the presence of a conceivable dose threshold be identified safely. A necessary prerequisite for the success of such an exercise is the availability of sufficiently accurate data in the low-dose range. The calculated response curves in Figure 1C may be used to assess the required data quality.

In discussing the results of Figure 1, I have concentrated on the dose scale. No attention has been paid to the question of whether the employed response parameter is properly reflecting the magnitude of inflammation induced by particle instillation. At low doses at which the number *n_PMN_* of generated PMNs is small compared with the number *n_mo_* of (lavageable) macrophages and other cells, it does not make a significant difference for the dose–response relationship whether the response is quoted as *n_PMN_*, as the ratio *r_P,m_* = *n_PMN_*/*n_mo_*, or as in Figure 1, as the fraction *f_P,m_* = *n_PMN/_*(*n_PMN_* + *n_mo_*) (unless one encounters the rare case that *n_mo_* differs markedly at low doses). If, however, the applied dose is sizable or large, that is, *r_P,m_* > 0.2, a distinction between the three measures of response becomes important. This is the case for the two highest response data in Figure 1, which gave rise to the conclusion that PMN production had saturated or tended toward saturation. The picture changes significantly if the PMN fractions are converted ratios according to the relation


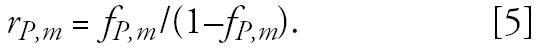


**Figure 2:**
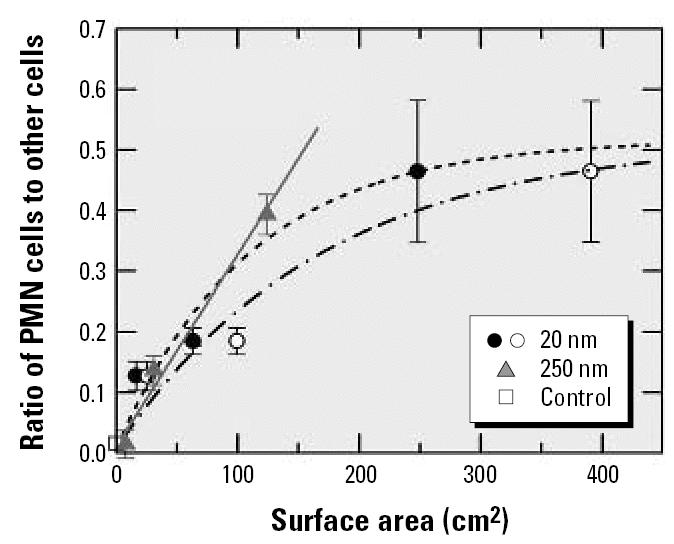
The same data as in Figure 1B but with the response quantified in terms of the ratio of the number of neutrophils to the number of macrophages and other cells. Data are mean ± SD.

The ratios thus obtained, which are analogous to those in Figure 1B, are shown in Figure 2. Within experimental uncertainty, all data for the 250-nm TiO_2_ particles (triangles) are now in accordance with the assumption of a linear dose–response relationship. The data for the 20-nm particles, on the other hand, are still compatible with Equation 1, as illustrated by the dashed and the dash–dotted lines. This evaluation shows that investigators must be concerned not only about the proper dose metric but also about the most adequate way of quantifying the response parameter.

**Figure 3:**
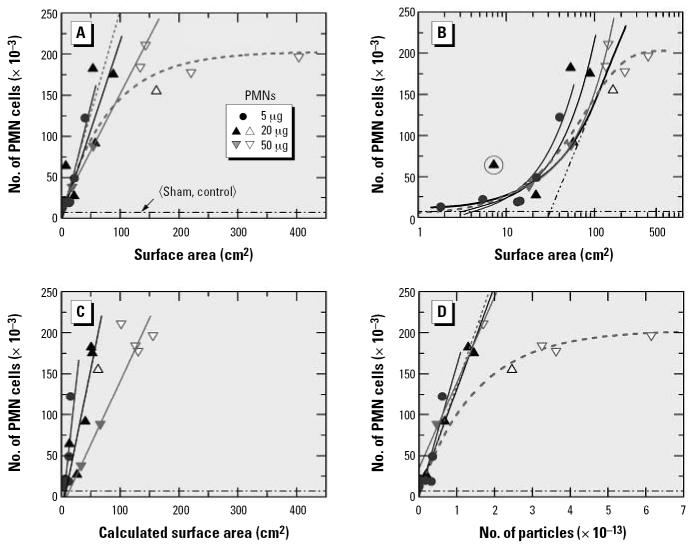
Neutrophil response of mice to instillation of six different types of carbon particles versus the BET-based surface area (*A,B*), the size-based surface area (*C*), and the particle number (*D*). Adapted from Stoeger et al. (2006). The dash–dotted straight horizontal lines in (*A–D*) represent the mean response observed in the sham and control experiments. Note the logarithmic *x*-scale in (*B*).

Considering the second set of data (Stoeger et al. 2006), Figure 3A and B shows lung inflammation data for exposure of mice to instillation of different types of carbon particles. The authors chose to quantify response in the most direct way—by the number of PMNs found in the BAL. In order to test the assumption that the inflammatory response scales with the surface area, I have distinguished the data by particle type. Merely for the ease of discussion, data for the same mass are identified by different symbols. Open and solid symbols of the same type relate to the same mass. Deviations from a linear response are clearly evident. The results suggest that the upper limit of linear response corresponds to a surface area of about 100 cm^2^. The data in the nonlinear dose range are denoted by open symbols. Selected by mass, the remaining data were passed through linear regression analyses. The results are represented by the three straight lines in Figure 3A. Clearly, the slopes are similar but not exactly the same; they decrease with increasing mass. This result could indicate that the upper limit of linearity is even lower than 100 cm^2^, more like 50 cm^2^. However, if that were the case, only one experiment (PrintexG particles with a surface area of 18 cm^2^) would fall into the linear dose range for a particle mass of 50 μg. By fitting Equation 1 to the high-dose data in Figure 3A, one obtains the thick dashed curve. The corresponding initial linear section is shown as a thin dashed straight line which, in support of the adequacy of the fit, agrees quite well with the linear fit through the data for exposure at a mass of 5 μg.

The data presented in Figure 3B, with the surface area on a logarithmic scale, was also used in the original publication (Stoeger et al. 2006), but the data were distinguished by particle type. The fit function used in the previous study is shown as a thick solid line. The authors stated that “regression analysis revealed a strong logarithmic relation for the surface area (*r*^2^ = 0.65).” This statement is problematic. Figure 3B shows that a logarithmic relation would correspond to a straight line, but in the original fit this is evident only for surface areas above about 80 cm^2^, that is, in the nonlinear dose range (see extrapolated thin straight dash–double dotted line). The apparent linearity at high doses on the logarithmic scale may have led the authors to conclude that their data support a “threshold” surface area of about 20 cm^2^. But, in an appropriate linear presentation of the low-dose data (Figure 3A), there is no evidence for a threshold. The shape of a linear exposure–response relationship with a threshold has been discussed in a recent review (Oberdörster et al. 2005).

In addition I note that the experimental data sometimes reveal the presence of significant outliers. An example relating to 20-μg PrintexG particles is circled in Figure 3B. This data point was excluded in the detailed data analysis described below.

Mass-to-surface area conversion may also be carried out according to Equation 3, using mean particle sizes 〈*D*〉 determined from the TEM data (Takenaka S, personal communication, 2006) and a graphite mass density of 2.15 g/cm^3^. The used 〈*D*〉 values agree to within better than 5% with the mean sizes derived from the upper and lower size limits quoted in the original publication (Stoeger et al. 2006). Different from the example discussed above with reference to the TiO_2_ data in Figure 1B, this approach did not provide proper scaling of the experimental data, as shown in Figure 3C. Note the large differences in the slopes of the straight lines representing the results of linear regression analysis (applied only to the data represented by solid symbols). The evaluation would also suggest the presence of small threshold doses that increase with increasing mass. Furthermore, with this kind of mass-to-dose conversion, the effect of saturation in response at high doses appears to be removed artificially.

The data in Figure 3C are useful because they show clearly that the success of searching for the proper dose metric depends critically on the chosen particle parameter. Hence, at this stage of data evaluation, one could have hoped that it would be rather easy to identify a single parameter that serves best as a dose metric. Assuming that the BET-based surface area is the parameter of choice, it came as a surprise that good scaling of the experimental data could be achieved by converting the mass *M* to the number *N* of particles as


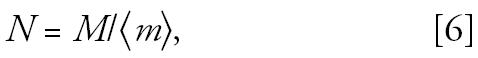


where 〈*m*〉 is the mean mass per particle,


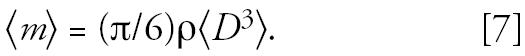


As shown in Figure 3D, the scaling with particle number is better than that with the BET-based surface area in Figure 3A, at least for the response data being studied. There is good agreement between the results in Figure 3A and D in that essentially the same data points are identified as falling into the nonlinear dose range (assignment uncertain for one data point located near the assumed border line between the linear and the nonlinear sections).

**Figure 4:**
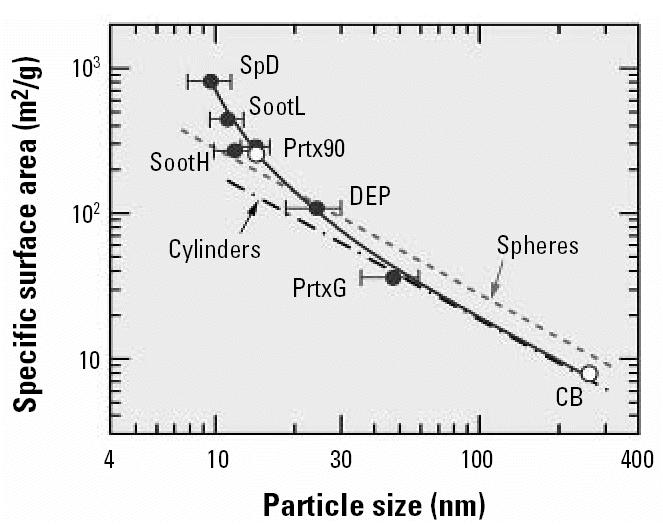
BET-based specific surface area versus the particle size by TEM. Solid circles (mean ± SD) adapted from Stoeger et al. (2006); open circles are adapted from Donaldson et al. (2000). Surface areas calculated for compact particles are shown for comparison as dashed and dash–dotted lines.

To explore the the observation that good scaling can be achieved using two different particle parameters, I first considered the relation between the specific BET surface areas (Stoeger et al. 2006) and the mean particle size shown in Figure 4 as solid circles. Two additional data points, shown as open circles (CB denotes carbon black) from another study (Donaldson et al. 2000), fit well into the solid-circle trend. The specific surface areas according to Equations 3 and 4 are denoted by dashed and dash–dotted lines (labeled “spheres” and “cylinders,” respectively). The BET data agree with results obtained for compact particles (spheres) with sizes around 20 nm, but the smaller the particle size the more strongly the BET data exceed the predictions for spheres. This find-ing could indicate that the small particles are highly porous, notably those particles produced in a spark discharge (SpD) [the notation “ultrafine carbon particles” (ufCP) used by Stoeger et al. (2006) for spark discharge particles was not adopted here because all six types of particles investigated by the previous authors were ultrafine carbon particles].

An alternative explanation for the large BET surface area could be that the spark-generated matter contains a significant fraction of very small particles (flakes or chunks) that have escaped detection in TEM analysis. Supporting evidence for this supposition has recently been obtained in studies on spark-generated iridium particles analyzed by secondary ion mass spectrometry (Szymczak et al. 2006). For particle sizes > 20 nm, the BET data in Figure 4 are lower than calculated, approaching a ratio of 0.67 for particle sizes > 50 nm. In this size range the BET data formally correspond to the results calculated for cylindrical particles (Equation 4). Note that in the case of TiO_2_, the trend was just the opposite: the 250-nm particles suggest the presence of spheres, and the 20-nm data were in accordance with the assumption of cylindrical particles.

Regarding the two equally useful dose metrics, I compared the parameters of interest in reduced form. Similar to the specific surface area, I define the specific particle number *N_s_* as the number-to-mass ratio of compact particles:


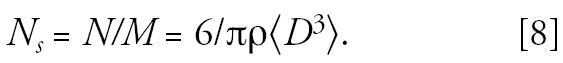


**Figure 5:**
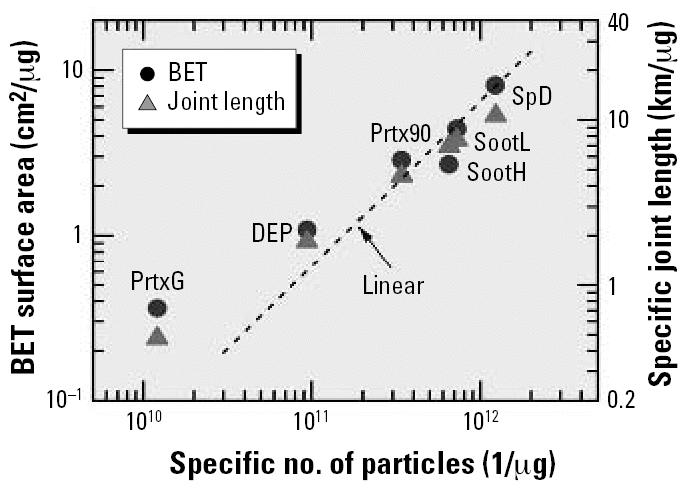
BET-based specific surface area (left scale) and specific joint length (right scale) of carbon particles versus the specific number of particles.

The relation between the BET surface area and *N_s_* is shown in Figure 5 (solid circles). The data for the four smallest types of particles—Printex 90 (Prtx90), flame soot with low and high organic carbon content (SootL and SootH, respectively) and SpD—scatter around the dashed straight line, which reflects direct proportionality between the BET surface area and the particle number. This proportionality is the reason that both parameters serve equally well as dose metrics. The deviation from linearity for the larger two types of particles, diesel exhaust particles (DEP) and PrintexG (PrtxG), does not play a large role because they feature small surface areas and small particle numbers and produced only rather small responses in the exposure studies.

The BET surface areas in Figure 4 roughly scale as 1/*D*^2^. This observation suggests that there is some kind of correlation with the (mean) joint length, 〈*L*〉 = *N*〈*D*〉, of particles (Wittmaack 2002). The specific joint lengths, *L_s,_* defined as


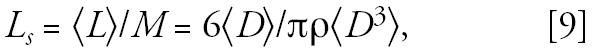


are also plotted in Figure 5 as a function of *N_s_* (solid triangles, right scale). In quantitative terms, *L_s_* ∝ *N_s_*^2/3^; that is, the deviation from linearity is again small for the four smallest yet most important types of particles, which, among each other, differed in size by a factor of < 1.5. Hence, one can expect that the joint length will be a third possible dose metric for the data under consideration. Evidence is presented later in this article.

**Figure 6:**
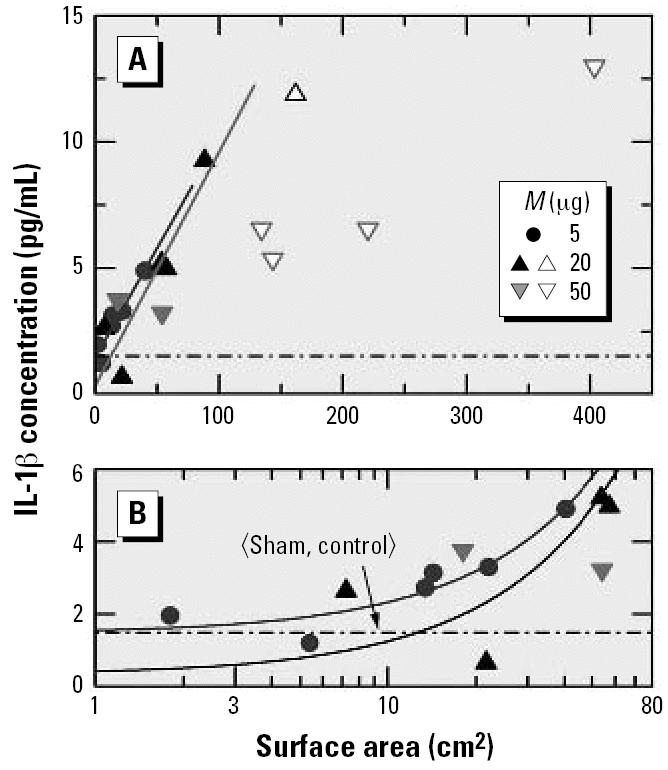
BALF IL-1β response of mice to instillation of six different types of carbon particles versus the surface area: (*A*) linear scale and (*B*) logarithmic scale. Adapted from Stoeger et al. (2006).

**Figure 7:**
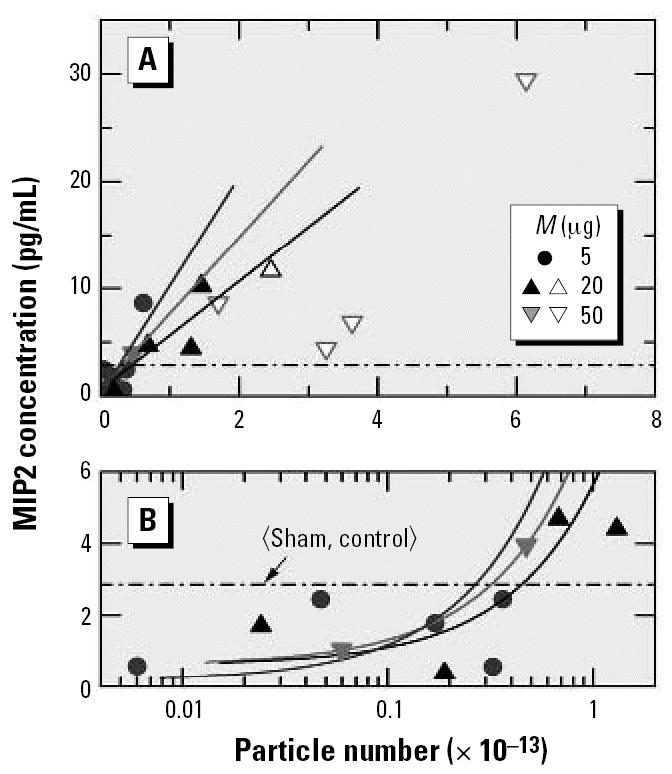
BALF MIP2 inflammatory protein response of mice to instillation of six different types of carbon particles versus the particle number: (*A*) linear scale and (*B*) logarithmic scale. Adapted from Stoeger et al. (2006).

Until now I have considered only the results for PMNs. Data for the inflammatory response derived from the concentrations of IL-1β and MIP2 in BALF are presented in Figures 6 and 7, respectively. In Figure 6 the surface area was used as a dose metric and in Figure 7 the particle number was used. In contrast to the PMNs (Figure 3), the cytokine concentrations in Figures 6A and 7A do not exhibit a clear saturation in response, which means that the data cannot be described in a satisfactory manner by Equation 1. The result could indicate that the response rate was changing when going from low to high doses, which could also provide an explanation for the observation that the high-dose cytokine data exhibit much larger scatter than the PMN data. When linear regression is applied rigorously to the low-dose results for 5 and 20 μg, the slopes of the resulting straight lines in Figures 6A are almost the same, as one would expect in the linear dose regime. The two low-dose data points for 50 μg, on the other hand, do not yield a meaningful linear relation, which provides additional evidence of large statistical fluctuations in the measured response. Presumably these fluctuations are also responsible for the sizable differences in the slopes of the low-dose linear regression lines in Figure 7A.

In Figures 6B and 7B, the low-dose response data for IL-1β and MIP2 are also presented on logarithmic dose scales, which is to illustrate that sufficiently detailed measurements at low doses provide a better means of identifying the zero-dose response (or offset) than using a rather limited number of sham or control experiments. This is particularly evident from Figure 7B, in which as many as eight data points fall below the mean of the sham and control experiments. These data indicate that experiments of the kind discussed here are meaningful only if they are performed carefully in the low-dose region. Only with such data at hand can one determine the two parameters of interest, that is, the response rate *R_m_*/*M_c_* and the offset *R*_0_ defined in Equation 2. Separate control experiments are meaningful only if they are statistically highly significant. Because of established ethical restrictions on the number of animals available for one particular study, low-dose experiments should be given preference to control experiments.

**Figure 8:**
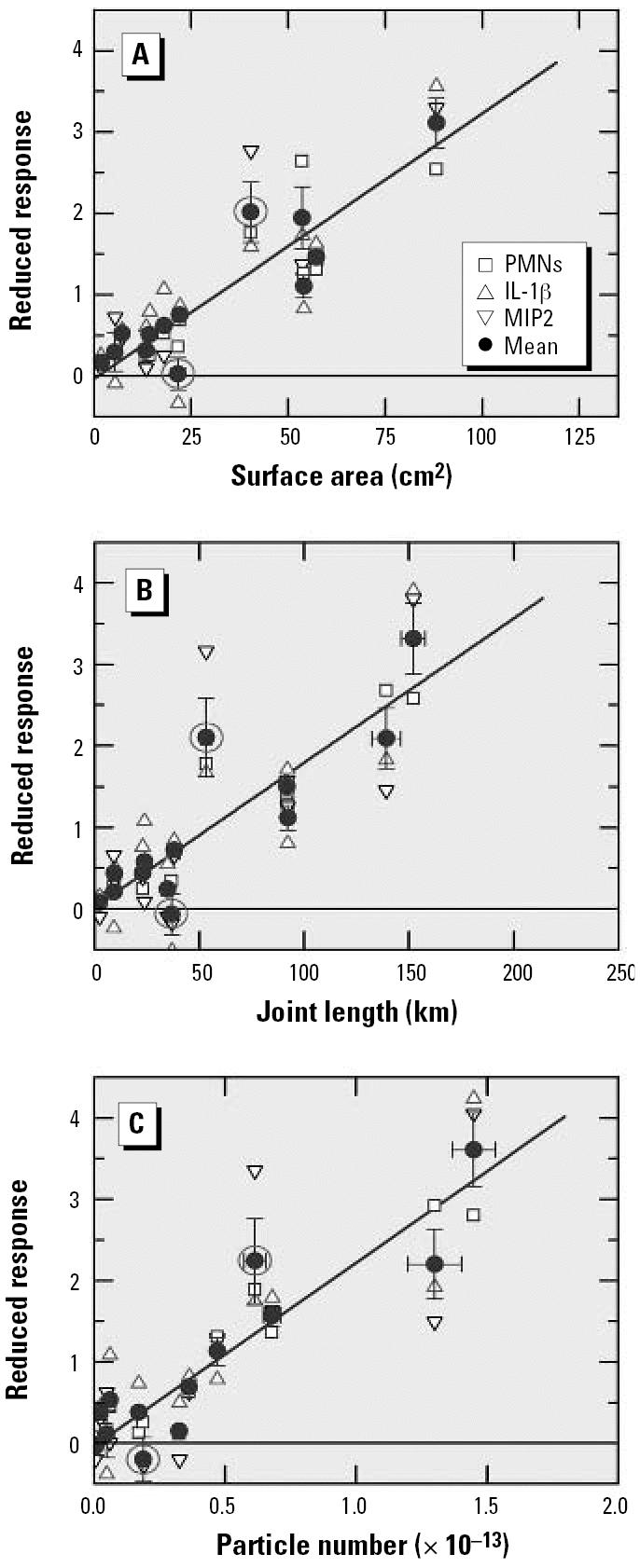
Reduced inflammatory response versus different dose parameters: (*A*) BET surface area, (*B*) joint length, and (*C*) particle number. Mean off-set *R*_0_ subtracted from the response data. Data are mean ± SD.

Considering the results of Figures 3A, D, and 6A, one could conclude that the particle number and the surface area are both suited as a dose metric. In other words, all response data obtained at low doses may be combined, regardless of mass, to yield one data set that allows *R_m_*/*M_c_* and *R*_0_ to be determined with a statistical significance that will be markedly improved compared with the analysis of data for one response parameter only. In the actual evaluation, 12 low-dose data points were included in the PMN set, 13 each in the IL-1β and the MIP2 sets. The data are presented in Figure 8 as a function of three dose metrics: the BET surface area, the particle number and, additionally, the joint length (the large amount of particulate matter instilled at the upper end of the linear dose range is evident from the abscissa in Figure 8B, which shows that the data extend to joint lengths of > 150 km). Linear regression analysis of the data in Figure 8 yielded nine sets of results for *R_m_*/*M_c_* and *R*_0_ as well as the respective values for *r^2^* and *p*. The statistically most significant results were obtained for PMNs, with *r^2^* between a low for the surface area (0.79) and a high for the particle number (0.91) and *p* < 10^−4^ throughout. The MIP2 data produced statistically least satisfactory results, ranging from *r^2^* = 0.53 (*p* = 5 × 10^−3^) for the length to *r^2^* = 0.69 (*p* = 4.4 × 10^−4^) for the surface area. After subtracting *R*_0_ from the respective data set, the off-set-corrected responses were divided by the mean to determine 3 × 3 sets of scaled response data *R_i,_*_φ_ for response type *i* and dose φ. The results are shown in Figure 8A, B, and C as open symbols. The three sets of data in each panel were finally weighed with *r^2^* and then used to calculate the mean 〈*R*〉_φ_ and the SD of the mean 〈Δ *R*〉_φ_. Several conclusions can be drawn from the results in Figure 8:

*a*) The scaled responses *R_i,_*_φ_, with *i* representing PMNs, IL-1β, and MIP2, agree quite well. Even the most extreme “outliers” do not deviate by more than 2〈Δ*R*〉_φ_ from 〈*R*〉 _φ_. None of the *R_i,_*_φ_ values exhibit an unusual trend as to the sign of the deviations from 〈*R*〉_φ_. The deviations appear to be solely statistical in nature. This implies that the relative magnitude of the response initiated by exposure to the ultrafine carbon particles is the same for all three types of responses that were used to explore lung inflammation on the basis of cellular and molecular matter found in BAL.

*b*) Linear regression analysis of the 〈*R*〉_φ_data yielded the straight lines shown in Figure 8A–C. The corresponding *r^2^* values were 0.76 (joint length), 0.82 (BET surface area) and 0.84 (particle number), all with *p* ≤ 10^−4^. Several of the 〈*R*〉_φ_data exhibit statistically significant deviations from the straight lines. Two examples are encircled. The positive deviation is due to SpD (5 μg) and the negative is due to DEP (20 μg). If these two data and 〈*R*〉_φ_ for DEP (50 μg; not highlighted) are excluded from the regression analysis, the resulting *r*^2^ values improve significantly, but the “order” of the dose parameters changes: 0.90 (particle number), 0.92 (joint length), and 0.96 (BET surface area). The important consequence of these two sets of regression analyses is that I cannot safely identify one dose parameter as serving best for quantifying the magnitude of lung inflammation due to instillation of carbon nanoparticles.

**Figure 9:**
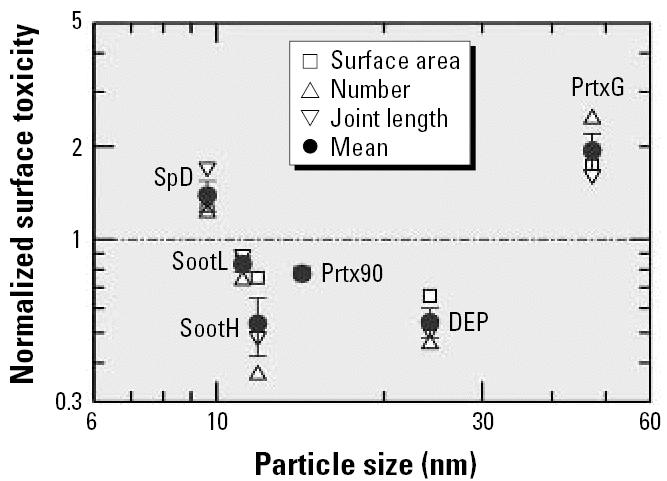
Normalized surface toxicity versus particle size. Solid circles are mean ± SD.

*c*) The observation of statistically significant deviations from the mean suggests that one of the fundamental assumptions on which data evaluation was based might not be valid, the assumption that different types, *j*, of carbon particles feature the same surface toxicity. To explore this aspect further, relative surface toxicities τ*_j,k,x_* were defined as


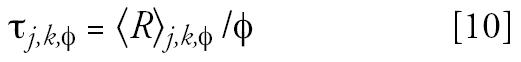


separately for the three dose parameters *k* (surface area, joint length, and particle number) and for all φ in the low-dose limit. Relative surface toxicities τ*_j,k_* were obtained as the mean of the τ*_j,k,_*_φ_-data available for different φ at the same combination of *j* and *k*. Normalized surface sensitivities, τ*_j,k_^n^*, defined as


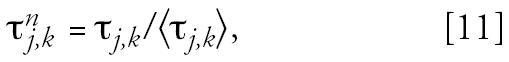


are depicted in Figure 9 as open symbols. The mean of these data for each particle type (solid circles) constitutes the normalized surface toxicity τ*_j_^n^*. Two types of particles, SpD and PrtxG, are well above the mean of unity, and two other types, SootH and DEP, are well below. The consequence of the observed pronounced differences in surface toxicity is that particles made of the same material but by different techniques are not generally suited for identifying the proper dose metric in studies designed to determine the effect of particle size. Hence, unless one can find particles of the same material and with sufficiently similar surface toxicity, but with distinctly different physical parameters such as size and surface area, it will be difficult to determine which of the physical parameters is best suited as a dose metric.

The origin of the differences in surface toxicity is not clear at this point. We note that diesel exhaust particles are at the low end of surface toxicity. This finding should be important in the ongoing discussion on adverse health effects due to traffic-related emissions. The low toxicity of DEPs may provide an explanation for the reported lack of concordance between lung cancer risk levels and diesel exhaust exposure (Valberg and Watson 2000).

SootH and DEP particles were similar in terms of low surface toxicity; both were characterized as containing about 20% organic carbon (OC; Stoeger et al. 2006). One could argue that the presence of OC, presumably “on” the particles, reduces the surface toxicity compared with the “naked” particles. However, the SpD particles contained almost the same OC fraction (17%) as SootH and DEP, but the surface toxicity of the former is high according to Figure 9. This apparent controversy could be resolved by arguing that without OC these particles might have exhibited an even higher toxicity than observed, possibly because of the presence of small chunks and flakes discussed above. Another argument in favor of differences in surface toxicity is that in contrast to all the other types of carbon particles discussed here, the SpD particles were not a product of incomplete combustion. Hence, they could feature a morphology distinctly different from that of the other particles. This is one of the issues that needs to be clarified by HRTEM investigations or by other advanced methods of particle characterization.

**Figure 10:**
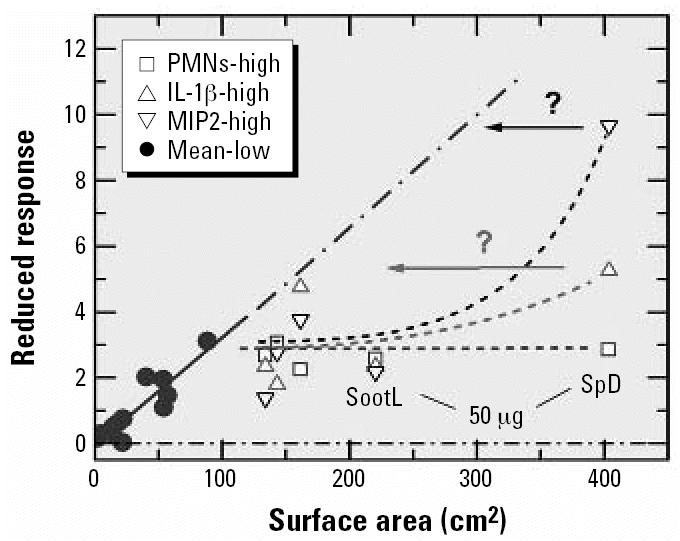
Reduced inflammatory response versus the BET surface area, with emphasis on the data at high doses. The arrows and the associated question marks denote the large correction in dose that may be required to account for the effect of conceivable compaction of the very small spark-discharge particles, notably in the high-mass instillation experiments.

Returning to the problem of nonlinear response, it is tempting to apply the low-dose normalization factors to the high-dose data as well. The results of this exercise are presented in Figure 10. Whereas at low doses only the mean values of Figure 8 are reproduced, the high-dose data are shown separately for all three response parameters. Presenting the data in this manner allows the large differences in response at high doses to become evident. The results support the notion, which is already suggested by the data evaluation presented in Figure 1B, that the use of high-dose data for identifying the proper dose metric will give rise to misleading conclusions.

The data in Figure 10 should be interpreted carefully. Apart from suggesting a reduced response rate at excessively high doses, the data could also be read as providing evidence that, beyond some limit, conversion from mass to a certain dose parameter is not correct any more. For SpD, the highest doses correspond to a number concentration of 1.2 × 10^15^ cm^−3^, or a joint length of 10^4^ km/cm^3^, in the instilled solution. At these large concentrations one should expect to encounter very heavy coagulation or compaction of particles, notably in the case of those small particles that are contained in the SpD and SootL matter. Compaction would mean that the “true” dose in terms of particle number, surface area, or joint length was much lower than calculated for individual particles. Hence, the response data should be shifted to significantly lower doses, as indicated by the arrows in Figure 10. This uncertainty in dose assignment constitutes yet another argument against high-dose experiments or, alternatively, an argument for improved methods to ensure adequate particle dispersion during instillation (Rao et al. 2003).

**Figure 11:**
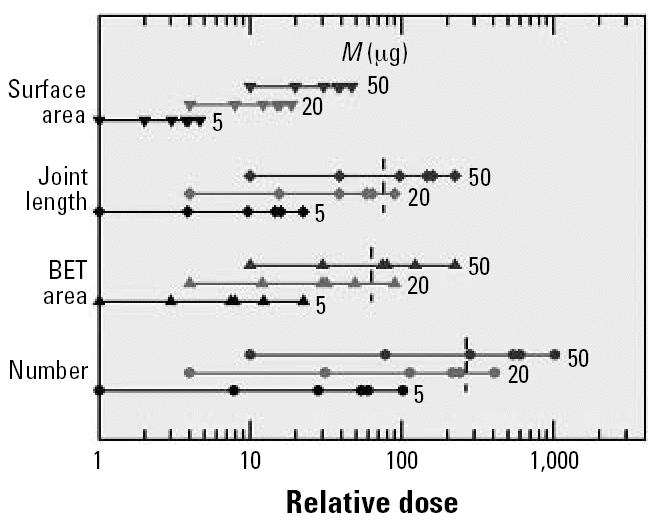
Illustration of the relative dose ranges covered by the four different dose parameters used for data evaluation. The vertical short dashed lines indicate the limit up to which the dose–response relationship is linear within experimental accuracy.

The question should also be addressed as to why three different particle parameters were suited as dose metrics but not the surface area calculated under the assumption of compact particles. The formal answer to this question can be found in the data presented in Figure 11, which shows the range of relative doses covered by the four different particle parameters. For ease of comparison, the data are normalized to the lowest dose, namely, to 5 μg PrtxG. The large differences in dose range are evident. Considering the dose parameters derived from the size *D* of the particles, the differences are solely due to the fact that mass-to-dose conversion is basically proportional to *D*^−s^, with the power *s* increasing from 1 through 2 to 3 for the surface area, the joint length, and the particle number, respectively. Accordingly, the corresponding maximum relative dose increases from 47 through 220 to 1,020, each time by a factor 4.7, which equals the ratio of the largest to the smallest particle size. The most appropriate dose metric is the particle parameter that provides optimum “stretching” of the dose range in terms of mass. According to the data presented in Figure 3C, dose stretching is not sufficient using the size-based surface area as a dose metric. To achieve optimum scaling, one has to stretch the dose scale by another factor 22 (= 4.7^2^); that is, one has to use the particle number as a dose metric, as illustrated in Figure 3D. For the PMN data discussed in Figure 3A, the BET-based surface area provides reasonable but not optimum stretching. This is also true for the joint length, in which case Figure 11 indicates that the quality of scaling is almost the same as for the BET surface area (results not shown in Figure 3).

Even though Figure 11 provides the basic rationale for selecting the proper dose metric, there are several reasons why a final decision in favor of a “best choice” is not yet possible. First, the statistical uncertainty of the response data is too large. Second, the observation of particle-type dependent surface toxicity introduces an uncertainty that cannot be resolved at present. Third, the particle parameters and their statistical variation within a sample are not known well enough to safely convert the particle mass to a conceivably appropriate dose metric.

## Conclusion, Further Comments, and Prospects

This present study has addressed several issues and problems in the field of ultrafine particles and lung inflammation. The need for linear dose–response studies has been demonstrated. Such studies are possible, not only by investigating a single but also multiple response parameters. Another problem is the large differences in the surface toxicity that one may encounter with particles made from the same material but by different techniques. Such differences aggravate or even exclude the identification of the proper dose metric, if such a parameter exists at all. In fact, there is no reason to assume that the contribution of the physical properties of a particle to the observed inflammatory response can be fully described in simple terms such as the size or the surface area.

One of the most important issues concerning experiments with laboratory-generated carbon particles relates to the question of whether the aggregates formed during the production process will remain intact or will dissociate after deposition in the lung (Wittmaack 2006). In this context it is important to note that the particle sizes determined by TEM (Stoeger et al. 2006) were derived by the analysis of rare individual species identified on the TEM support grid (Takenaka S, personal communication, 2006), the reason being that size evaluation within chain aggregates is difficult because of the frequent overlay of particles. The observation that the particle number constitutes a good if not the best dose metric for the response to carbon particles may be considered a strong indication that the aggregates did in fact dissociate in the lung before causing inflammation. Diesel soot aggregates constitute an exception. Many (probably even most) of the individual particles that one can identify in TEM (Bérubé et al. 1999) and SEM (Wittmaack 2004) images were tightly bound together during growth; that is, the crystallites observed at the neck between two adjacent primary particles extend through the neck, as shown by HRTEM (Ishiguro 1997). This state of tight aggregation may be the most important reason for the low surface toxicity of diesel soot.

It must be kept in mind that *in vivo* studies involving animal exposure to particulate matter can be justified only if there is a good chance for improving current knowledge on the response of humans to inhaled ambient aerosol particles. Even though the toxic potential of ambient PM is too low to make adverse health effects plausible (Green et al. 2002; Valberg 2004), this aspect should not remain the most powerful argument in support of the speculation that ultrafine particles cause the problem. Arguments for the ultrafine particle hypothesis can be misleading. Oberdörster et al. (2005) and Nel et al. (2006) pointed out, for example, that the surface area-to-mass ratio *A*/*M* of particles increases with decreasing particle size, as discussed with reference to Equation 3. In toxicologic studies, however, it is believed that *A* not *A/M* is important—provided the suface area is the proper dose metric. Previous reasoning (Nel et al. 2006; Oberdörster et al. 2005) was based on the inherent assumption that the mass concentrations of nanoparticles is reasonably constant over a wide range of particle sizes. Typical size distributions of ambient PM tell a completely different story. At urban sites it is the (mean) number rather than the mass concentration that is roughly constant between 20 and 200 nm (Wittmaack 2002), so that *A* decreases strongly with decreasing *D,* as π*D*^2^*N*. Hence, when referring to *A*/*M* rather than to *A* as the quantity of concern, the hazard due to ambient nanoparticles is exaggerated by a factor proportional to 1/*D*^3^. In view of these facts I must conclude that associations between adverse health effects and ambient PM are implausible with respect to both the chemical composition (mass) and the surface area of the particles. Unraveling this mystery is a challenge to scientists active in environmental medicine, toxicology, biophysics, and epidemiology.

To illustrate the need for refinement of current research, one example may suffice. The size distributions of ambient PM typically feature a fall-off in number concentration at particle sizes below about 20 nm, to the end that the concentrations between 5 and 10 nm may be an order of magnitude lower than between 20 and 200 nm (Wittmaack 2002). Therefore, experiments involving particles with sizes of ≤ 10 nm are of rather questionable relevance with respect to environmental health issues. Future *in vivo* studies should focus on carbon particles in the size range between 20 and 100 nm, including diesel soot. In such investigations the challenge will be that these particles produce only a comparatively low inflammatory response, as discussed in detail above.
